# The Buffalo State Asylum for the Insane

**Published:** 1880-08

**Authors:** 


### THE BUFFALO STATE ASYLUM FOR THE INSANE.

THE Board of Managers of the Buffalo State Asylum for the Insane have wisely chosen for the responsible position of Superintendent, Dr. Judson B. Andrews, of Utica, a gentleman of extensive experience, of wide culture, and rare administrative ability, to organize and put in successful operation the State institution located in this city. We unite in the sentiment so generally expressed throughout the State, of approval of this excellent selection. The Buffalo Asylum, under his able management, will at once assume the leading position among the institutions for the care of the insane in this country. We do not fail to recognize also the important accession to our professional circles, secured through the removal of Dr. Andrews to our city. The Buffalo Medical College have in their announcement notified the profession of the addition to its curriculum of a course of lectures on nervous diseases, by Dr. Andrews. This is a most important addition to its lecture course, and will greatly add to the superior advantages already offered in our city, in the matter of medical education.

